# Sagging Eye Syndrome or Nemaline Rod Myopathy? Divergence Insufficiency with Levator Dehiscence as an Overlapping Symptom between Two Diagnoses

**DOI:** 10.1155/2017/1708734

**Published:** 2017-01-15

**Authors:** Stephanie S. L. Cheung, Larissa K. Ghadiali, Thomas H. Brannagan III, Gul Moonis, Phyllis L. Faust, Jeffrey G. Odel

**Affiliations:** ^1^Hospital Authority, Hong Kong; ^2^Department of Ophthalmology, Columbia University Medical Center, New York, NY, USA; ^3^Department of Neurology, Columbia University Medical Center, New York, NY, USA; ^4^Department of Radiology, Division of Neuroradiology, Columbia University Medical Center, New York, NY, USA; ^5^Department of Pathology and Cell Biology, Columbia University Medical Center and the New York Presbyterian Hospital, New York, NY, USA

## Abstract

A 78-year-old woman complained of gradual, painless onset of horizontal binocular diplopia associated with progressive axial weakness. Physical examination revealed esotropia that was greater at distance than at near vision, bilateral levator dehiscence, and normal abducting saccadic speeds. Given the age of the patient and compatible clinical findings, the diagnosis of Sagging Eye Syndrome (SES) was made. However, further work-up with a muscle biopsy suggested Sporadic Late-Onset Nemaline Myopathy (SLONM) as the cause of her progressive muscle weakness. Although rare, external ophthalmoplegia has been described in the literature as a presenting symptom in SLONM. To elucidate the pathological mechanism for the patient's diplopia, an MRI of the orbits was performed, which revealed findings consistent with SES. This case aims to highlight the importance of integrating clinical findings during the diagnostic process and serves as a reminder that diplopia can be a common symptom for an uncommon diagnosis.

## 1. Introduction 

Sagging Eye Syndrome (SES) is a recently recognized syndrome that involves age-related degeneration of orbital connective tissue, resulting in mechanical strabismus due to thinning and ultimately separation of the lateral rectus-superior rectus intermuscular septum [1]. The recognition of this syndrome has reduced the need for imaging and further neurological investigations in the setting of divergence insufficiency in the aging population. Here, we describe an unexpected diagnostic conundrum: an elderly woman complaining of diplopia presented with a clinical picture compatible with the diagnosis of SES; however, additional chronic systemic symptoms necessitated a muscle biopsy, which revealed a rare form of myopathy. Ultimately, imaging was needed to elucidate the cause of this patient's ocular symptoms. This case demonstrates the role of imaging in diagnosing neuroophthalmological conditions, as well as highlighting diplopia as a common symptom for an uncommon diagnosis.

## 2. Case Report 

A 78-year-old woman with a history of a remote right-sided cerebrovascular accident complained of painless, progressive onset of binocular horizontal diplopia, elicited by switching from near to distance vision. Prism glasses improved her symptoms. In addition, she had noticed progressive onset of axial weakness, dysphagia, and imbalance three years earlier, which had significantly worsened in the past few months as the ocular symptoms presented. Her symptoms progressed until she was unable to support herself when sitting and walking and required the use of two canes to ambulate.

On examination, her distance visual acuity was 20/20 OU. There was no dyschromatopsia or defects on Amsler grid testing. Pupils were equal and briskly reactive to light without a relative afferent pupillary defect. Trigeminal and facial nerve functions were intact. Her marginal reflex distance (MRD1) measured 3 mm in the right eye and 2 mm in the left. Levator function was 14 mm in both eyes. Ocular motility exam revealed comitant esotropia of 12–15 PD at distance vision and smaller esophoria of 6 PD at near vision. Extraocular movements were full and saccades, smooth pursuit, and OKN were intact. There was no pathological nystagmus. Slit lamp examination revealed bilateral map dot fingerprint dystrophy. Funduscopic examination was normal. Our clinical impression was divergence insufficiency and bilateral levator dehiscence due to Sagging Eye Syndrome.

**Figure 1:**
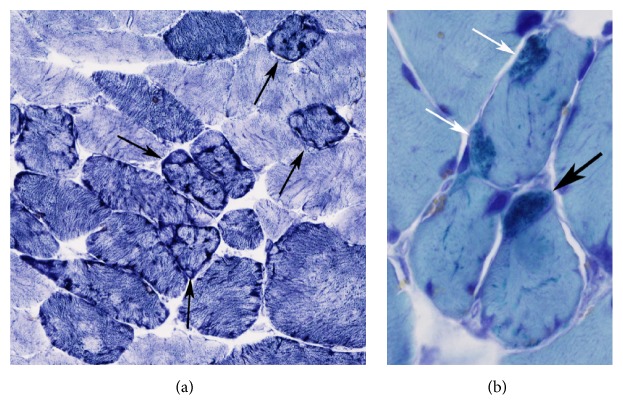
Pathologic findings in quadriceps muscle biopsy. (a) NADH stain. Chronic myopathic changes with fiber size variation and myofibrillar alterations, including many lobulated fibers (arrows). (b) Gömöri trichrome stain. Many dark staining nemaline rods are seen in a highly atrophic fiber (black arrow) and aggregated beneath the sarcolemma (white arrows) and scattered in sarcoplasm in these adjacent myofibers.

The patient was evaluated by Neurology for her axial weakness. In addition to her severe axial weakness, she had MRC grade 4 strength in the neck flexors, extensors, deltoids, and hip flexors. EMG revealed evidence of left lumbosacral radiculopathy at L2–L5 levels without electrophysiological evidence of length-dependent large fiber neuropathy, neuromuscular junction disorder, or myopathy. Studies for CK, HbA1c, TSH, IgG, IgA, IgM, SPEP, UPEP, vitamin B panel, paraneoplastic autoantibodies, ANA, ANCA, cryoglobulin, and autoantibodies for Celiac disease (Gliadin Pep Ab IgA and TTG IgA) and Sjogren's syndrome (SS-A 52, SS-A60, and SS-B Ab IgG) revealed no abnormalities. In view of a lack of obvious cause for the patient's muscle weakness, a muscle biopsy of the left quadriceps was performed. The biopsy showed chronic myopathic changes with many lobulated fibers and several fibers containing rods, suggesting an adult-onset nemaline myopathy (Figures 1(a) and 1(b)). A single fiber with rods was also detected in the deltoid muscle.

**Figure 2:**
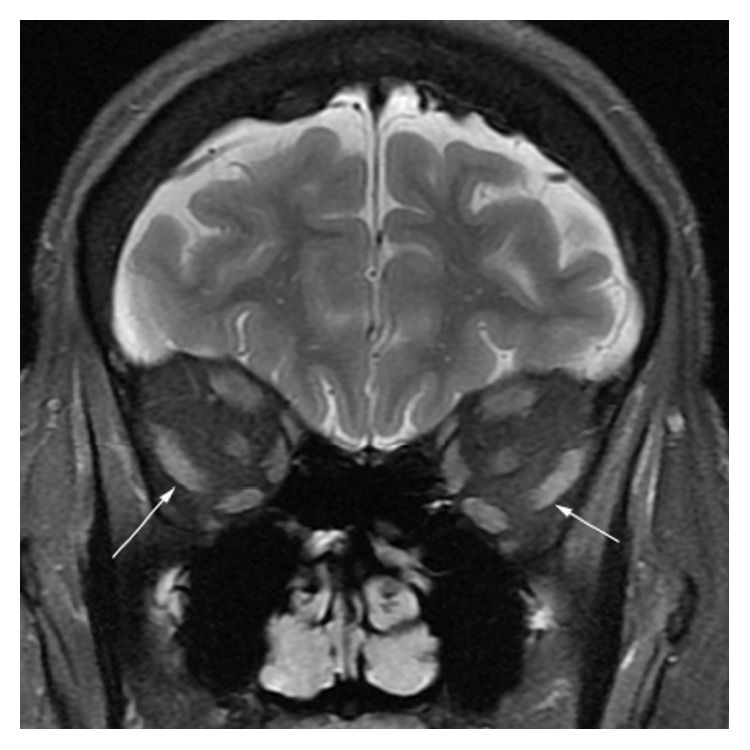
Coronal T2 weighted image through the orbit demonstrates inferior displacement of the lateral rectus muscles bilaterally (arrows) without abnormal signal.

An MRI of the orbit with and without contrast was performed to evaluate for SES versus a myopathic involvement of extraocular muscles. Imaging demonstrated inferior displacement of the lateral rectus muscle bilaterally (Figure 2). The muscles were normal in signal intensity without abnormal enhancement.

The patient was subsequently treated with IVIG and noted improvement in her limb weakness but thus far has not noted any improvement in core muscle weakness. Her diplopic symptoms were alleviated by prism lenses.

## 3. Discussion

Nemaline rod myopathies are a heterogeneous group of myopathies that are characterized by the presence of rod-like structures in skeletal muscle. Ocular symptoms are recognized as rare additional symptoms in congenital nemaline myopathy [2–4] but, since the description of the disease in 1963 by Shy et al. [5], there has only been one case of adult-onset nemaline myopathy with ophthalmoplegia as an initial manifestation [6]. Sporadic Late-Onset Nemaline Myopathy (SLONM) typically presents in patients older than 40 years of age with an unremarkable family history and a slowly progressive weakness of limb-girdle and axial muscles. Diagnosis is based on the clinical history of subacute evolving muscle weakness and the presence of nemaline rods on muscle biopsy. Myopathic EMG with fibrillation potentials and normal or low serum CK are also supportive of the diagnosis. A definitive diagnosis of nemaline myopathy requires histological proof of the presence of nemaline rods in muscles. Although nemaline rods are not pathognomonic for the disease, as they may be present as a minor feature in normal and aging muscle as well as a number of other inherited and acquired disorders, nemaline myopathy is diagnosed when nemaline rods are the predominant finding [7]. In this patient, the demonstration of chronic myopathic changes with a combination of nemaline rods and lobulated fibers in the quadriceps muscle supports the diagnosis of nemaline rod myopathy [8].

Sagging Eye Syndrome (SES), on the other hand, is a common finding in the aging population. Recently described by Chaudhuri and Demer [1], SES is associated with age-related orbital connective tissue degeneration, in which displacement of rectus pulleys and EOM elongation, associated with LR-SR band rupture, causes acquired vertical and horizontal strabismus. Patients with SES are recognized by their external appearances and motility patterns: levator aponeurotic dehiscence results in ptosis with a superior sulcus deformity and high eyelid crease, while rectus pulley displacements result in divergence paralysis esotropia (DPE) or cyclovertical strabismus (CVS). The establishment of SES as a clinical entity is significant in obviating the need for further neurological evaluation and imaging in patients presenting with acquired diplopia in the absence of neurologic complaints. In our patient, levator dehiscence and divergence insufficiency esotropia, along with full extraocular motility and normal saccades, were compatible with the diagnosis of SES.

Although the patient complained of axial weakness, imbalance, and dysphagia, these symptoms were long-standing and were initially attributed to her past history of CVA. SES was the most probable diagnosis when the patient's ocular symptoms were considered in isolation from her long-standing neurological deficit, but careful consideration of her clinical history suggested the possibility of a systemic cause for her divergence insufficiency. A muscle biopsy of the left quadriceps suggested an adult-onset nemaline myopathy, and it was not clear whether the eye findings were related to the myopathy or SES. In the end, MRI confirmed the initial diagnosis of SES. This case highlights imaging as an important diagnostic tool in neuroophthalmological conditions and also underscores diplopia as a common symptom with a long list of differential diagnoses.
